# Rapid glycoprotein evolution enables variant interactions in herpes simplex virus type 1

**DOI:** 10.1093/ve/veaf072

**Published:** 2025-09-17

**Authors:** Thomas Höfler, Michaela Zeitlow, Ji Y Kim, Emanuel Wyler, Jakob Trimpert

**Affiliations:** Institut für Virologie, Fachbereich Veterinärmedizin, Freie Universität Berlin, Robert-von-Ostertag-Straße 7, 14163 Berlin, Germany; Department of Diagnostic Medicine and Pathobiology, College of Veterinary Medicine, Kansas State University, 1800 Denison Avenue, Manhattan, KS 66506, United States; Institut für Virologie, Fachbereich Veterinärmedizin, Freie Universität Berlin, Robert-von-Ostertag-Straße 7, 14163 Berlin, Germany; Institut für Virologie, Fachbereich Veterinärmedizin, Freie Universität Berlin, Robert-von-Ostertag-Straße 7, 14163 Berlin, Germany; Department of Diagnostic Medicine and Pathobiology, College of Veterinary Medicine, Kansas State University, 1800 Denison Avenue, Manhattan, KS 66506, United States; Berlin Institute for Medical Systems Biology, Max-Delbrück-Center for Molecular Medicine in the Helmholtz Association, Hannoversche Straße 28, 10115 Berlin, Germany; Institut für Virologie, Fachbereich Veterinärmedizin, Freie Universität Berlin, Robert-von-Ostertag-Straße 7, 14163 Berlin, Germany; Department of Diagnostic Medicine and Pathobiology, College of Veterinary Medicine, Kansas State University, 1800 Denison Avenue, Manhattan, KS 66506, United States

**Keywords:** glycoproteins, viruses, evolution, herpes simplex, variant interaction, hypermutator, sociovirology

## Abstract

Glycoproteins cover the surface of enveloped viruses such as herpes simplex virus 1 (HSV-1). Whilst essential for cellular attachment and entry, they also are excellent targets for host immune responses. This dichotomy culminates in an evolutionary struggle in which receptor recognition and immune escape are intricately balanced. Herpesviruses feature a variety of different glycoproteins with diverse molecular functions. Here, we describe the rapid evolution of HSV-1 towards syncytial plaque phenotypes in Vero cell culture, as well as anti-gD antibody resistance in human foreskin fibroblast cells. Using a mild hypermutator virus to accelerate experimental evolution, we identified multiple genetic variants leading to syncytial plaques. Strikingly, these variants differentially affect interactions within viral populations. Whilst gK mutants engage in collective syncytia formation upon entry, accelerate superinfection exclusion and maintain fitness advantages at high multiplicities of infection, gB and gD mutants do not. Furthermore, we find gE mutants which lead to mouse anti-gD antibody resistance and cross protect wt virus in mixed populations. Our findings suggest complex social interactions within herpesvirus populations and illustrate the evolutionary plasticity and diverse function of their glycoproteins.

## Introduction

Glycoproteins constitute the outermost layer of enveloped virions (Flint *et al*. 2015, Howley *et al*. 2021). Herpesviruses like herpes simplex virus type 1 (HSV-1), encode a multitude of different glyco- and membrane proteins to ensure proper virion production and stability, as well as effective cellular attachment and entry (Agelidis and Shukla 2015, Eisenberg *et al*. 2012, Hilterbrand and Heldwein 2019). Specifically, HSV-1 encodes 12 glyco- and 5 membrane proteins. Glycoproteins are usually referred to by using a protein-based nomenclature which denotes gB, gC, gD, gE, gG, gH, gI, gJ, gK, gL, gM, and gN whilst membrane proteins are named according to their genetic locus, UL20, UL56, US9, UL24, and UL43 (Hilterbrand and Heldwein 2019). Together, these proteins form a dynamic and diverse viral envelope, important for interactions between viral particles as well as between virus and host cell. Essential for attachment and entry are 4 glycoproteins: gD, gH, gL, and gB (Cai *et al*. 1988, Eisenberg *et al*. 2012). Initially, gD recognizes one of the 4 cellular receptors, namely, nectin-1 and 2, herpes virus entry mediator and 3-O-sulfonated heparan sulphate (Campadelli-Fiume *et al*. 2000, Yoon and Spear 2004). Through conformational changes upon receptor binding, the entry signal is transmitted *via* the gH/L heterodimer to gB, which triggers membrane fusion (Atanasiu *et al*. 2010, Agelidis and Shukla 2015, Hilterbrand *et al*. 2021). Multiple other viral glycoproteins influence membrane fusion by diverse interactions with a variety of cellular receptors (Satoh *et al*. 2008).

Syncytia, multinucleated cells created by cell-to-cell fusion, play an important biological role, whether it is in skeletal muscles or the mammalian placenta (Huppertz *et al*. 2001). Some viruses also induce syncytia formation as a mean to increase cell-to-cell spread (Jessie and Dobrovolny 2021). Viruses capable of syncytia formation include respiratory syncytial virus (RSV), paramyxoviruses, severe acute respiratory syndrome coronavirus 2, human immune deficiency virus (HIV) and many more (Symeonides *et al*. 2015, Buchrieser *et al*. 2020, Gamble *et al*. 2021, Jessie and Dobrovolny 2021). Syncytia formation in viruses is especially important for cell associated viruses like RSV, for which it is directly linked to replicative fitness (Norrby *et al*. 1970, Krzyzaniak *et al*. 2013). Extensively studied are syncytia in HIV where they promote viral spread and particle production but also play a role for intersubtype recombination and diversification of HIV populations around the globe (Chowdhury *et al*. 1992, Steain *et al*. 2008, Han *et al*. 2022). However, syncytia also lead to increased apoptosis, which can decrease overall virion production (Ferri *et al*. 2000, Ma *et al*. 2021).

Since glycoproteins decorate the surface of virions, they are the primary targets of adaptive immunity (Corti and Lanzavecchia 2013, Pelegrin *et al*. 2015). Especially gD and gB epitopes are known targets of neutralizing antibodies, as preventing receptor binding or membrane fusion both prevent viral entry and stop infection (Lee *et al*. 2013, Hilterbrand *et al*. 2021). The dichotomy between receptor recognition, binding and cellular entry on one, as well as evading immune responses on the other hand, presents an enormous evolutionary constrain for glycoproteins (Geller *et al*. 2015, Thomson *et al*. 2021, Tenthorey *et al*. 2022). Many viruses bypass the immunological pressure by rapidly evolving numerous serotypes or by altering glycosylation patterns of surface proteins (Sommerstein *et al*. 2015, Tse *et al*. 2017).

Experimental evolution already allowed for *in vitro* selection of syncytial phenotypes in HSV-1 (Kuny *et al*. 2020), whilst the utilization of mild hypermutator viruses, enabled adaptation to non-permissive cells in Marek’s disease virus (Xing *et al*. 2022), and significantly sped-up antiviral resistance development in HSV-1 (Höfler *et al*. 2024).

Here we use hypermutator viruses to accelerate experimental evolution of HSV-1 glycoprotein variants. We observe diverse genotypes which share key phenotypes, however, we also report nuances to their respective behaviour. Additionally, we take note of phenotypes that suggest mutual and beneficial interactions within viral populations (Díaz-Muñoz *et al*. 2017, Sanjuán 2021, Leeks *et al*. 2023b). This study highlights the power of experimental evolution and defines HSV-1 populations as a diverse and interacting community rather than a congregation of clonal viruses.

## Material and methods

### Cells and viruses

Vero cells (ATCC CCL-81) and human foreskin fibroblast (HFF) cells (ATCC SCRC-1041) were propagated in Dulbecco’s modified eagle medium (DMEM, Pan Biotech) containing 10% foetal calf serum (FCS, Pan Biotech), 100 IU/ml penicillin G (Carl Roth) and 100 μg/ml streptomycin (Carl Roth) at 37°C and 5% CO_2_. All HSV-1 viruses presented in this study derived from the F-strains used in our former studies (Brunialti *et al*. 2023, Höfler *et al*. 2024).

### Viral reconstitution

Polyethylenimine (pei) transfection was used for viral reconstitution. In brief, a mixture of DNA (2–3 μg) and pei (12 μl, 1 mg/ml polyethylenimine, linear (mw 25 000); Polysciences) was diluted in 100 μl Opti-mem (Thermo Fisher Scientific). The reaction was incubated for 30 min at room temperature (RT) and afterwards mixed with 1 ml cell culture medium. A sub-confluent 6-well plate well of Vero cells was overlayed with the transfection DNA-Medium mix for 4 h and afterwards replaced by fresh medium. Plates were kept in culture till 70%–100% of cells showed cytopathic effects (CPE).

### Propagating of cells and virus

Viral stocks were prepared and passaged on Vero cells as described previously (Höfler *et al*. 2024). For propagation on HFF cells 100 μl of a 1:100 dilution was used to infect a confluent 5 cm cell culture dish (corresponding to a MOI of *ca.* 0.01). To select for antibody resistance, 1 μl of the stock solution (mouse-αgD clone E317, The Native Antigen Company, UK; 1 mg/ml) was used to obtain a final concentration of 200 ng/μl.

### Viral titer determination

Plaque assays were utilized as described previously (Höfler *et al*. 2024). In brief, 10-fold serial dilutions were prepared and 100 μl were incubated on single wells of confluent 24-well plates of Vero cells. Inoculate was replaced by semisolid overlay (2.5% colloid microcrystalline cellulose, Aldrich; in 1× DMEM, Biochrom; supplemented with 10% FCS, 100 IU/ml penicillin G, 100 μg/ml streptomycin and 0.15% sodium bicarbonate, Sigma Life Science). Once visible plaques formed, plates were washed twice with PBS and fixed with 4% formaldehyde for 20% at RT. For staining a 0.75% crystal violet solution was used.

### Plaque size assays

Around 100 pfu were utilized per sample to infect single wells of 12-well plates of either Vero or HFF cells. Wells were overlayed, incubated for 2 days, washed and fixed as described above, followed by permeabilization (PBS supplemented with 0.1% triton X-100) for 10 min and blocking (PBS containing 1% FCS) for 2 h at RT. Overnight incubation (4°C) of the first antibody (C_2_D_8_, 1:100 dilution in PBS + 1% FCS) (Borchers and Ludwig 1991) was followed by secondary antibody (goat-anti-mouse alexa fluor 568 conjugated, 1:3000 dilution in PBS + 1% FCS, Thermo Fisher Scientific) incubation for 2 h at RT. PBS washing steps were performed in between and after antibody incubations. Plaque pictures were taken at a Zeiss Axio Vert.A1 inverted fluorescence microscope with 100x magnification and analysed with NIH ImageJ 1.52n (Schneider *et al*. 2012). To wt p0 normalized plaque areas were converted to diameters.

### Viral growth kinetics

Multiplicities of infection (MOI) of 0.001 and 0.01 in 6-well plates or 10 in 24-well plates were prepared in triplicates for multi- and single-step growth curves respectively. For single-step, inoculum was removed after 1 h, washed and overlayed with fresh medium. Timepoints for stock preparation and titration (see above) were set after 12 h, 1 d, 2 d, 3 d, 4 d, and 5 d as well as 1 h, 3 h, 6 h, 12 h, and 24 h post infection.

### Serum neutralization tests

Resistance to neutralizing antibodies was measured by serum neutralization tests. 2-fold serial dilutions of the antibody (mouse-αgD clone E317, The Native Antigen Company, UK) were prepared in 96-well plates and mixed with 200 pfu of each respective virus. After 1 h incubation at 37°C and 5% CO_2_, the antibody-virus mixture was transferred to a confluent 96-well plate of Vero cells. Three to four days later, plates were fixed and crystal violet stained as described earlier. Affected areas were measured using NIH ImageJ 1.52n (Schneider *et al*. 2012) and used for IC_50_ calculation following a non-linear model:


(1) \begin{equation*} \frac{f_a}{f_u}={\left(\frac{IC_{50}}{d}\right)}^m \end{equation*}

With *f_a_* and *f_u_* being the affected and unaffected fraction respectively, *d* being the antibody concentration and m the magnitude.

### Plaque reduction assay

Susceptibility to foscarnet was measured by plaque reduction assay as described previously (Höfler *et al*. 2024).

### Competition assays

Competition assays were performed as described previously (Höfler *et al*. 2024), *via* quantitative polymerase chain reaction (qPCR) for evolved HFF populations and *via* fluorescently labelled reporter viruses for p0 isolates. Primers for qPCR can be found in Supplementary Table 1.

### Particle stability tests

Viruses were diluted in DMEM supplemented with 10% FCS, 100 IU/ml penicillin G and 100 μg/ml streptomycin to titers of 10^5^ pfu/ml in ventilated 13 ml plastic tubes at 37°C and 5% CO_2_. Samples were titrated as described above every day for 5 days.

### DNA isolation

DNA for sequencing was isolated by a micrococcal nuclease extraction protocol (Volkening and Spatz 2009), as described previously (Brunialti *et al*. 2023). To exclude fragmented chromosomal DNA (< 3000 bp) polyethylen glycol (PEG) based size exclusion was performed (Clarke *et al*. 2014) as described previously (Höfler *et al*. 2024). DNA for qPCR was isolated with the innuPREP virus DNA/RNA kit (Innuscreen) according to the manufacturer’s instructions. Bacterial artificial chromosom (BAC) DNA was isolated from 200 to 500 ml of *Escherichia coli* overnight cultures using Qiagen’s MidiPrep kit.

### Next generation sequencing and bioinformatics

Sampled populations were sequenced on the Illumina MiSeq platform as described earlier (Höfler *et al*. 2024). FASTQ files are available at NCBI SRA, BioProject accession number PRJNA927130. Sequencing reads were analysed with Trimmomatic v0.39 (Bolger *et al*. 2014), Burrows-Wheeler aligner v0.7.17 (Li and Durbin 2009), Samtools v1.10 (Danecek *et al*. 2021), BCFtools v1.11, and LoFreq v2.1.3.1 (Wilm *et al*. 2012). For further information see https://github.com/hoeflet/antiviral-resistance-evolution.git. Non-synonymous to synonymous substitution rates (dN/dS) were calculated by normalizing nucleotide replacements to per site rates (dN and dS), multiplied by allele frequency and summarized per gene:


(2) \begin{equation*} {\frac{dN}{dS}}_{gene}=\frac{\sum_{i=1}^n\frac{AF_{N,i}}{c_{N,i}}}{\sum_{i=1}^m\frac{AF_{S,i}}{c_{S,i}}} \end{equation*}

With AF_N_ and AF_S_ being the allele frequency of a non-synonymous and synonymous mutations, respectively, as well as c_N_ and c_S_ being how often a mutation at that nucleotide site leads to an amino acid change or not (1 ≤ c_N_, c_S_ ≥ 3).

Alphafold3 predictions were performed by using Alphafoldserver.com (Abramson *et al*. 2024) and visualized with PyMOL (Retrieved from http://www.pymol.org/pymol). Principal component analysis was performed with a custom python script (https://github.com/hoeflet/antiviral-resistance-evolution.git) as described previously (Höfler *et al*. 2024).

**Figure 1:**
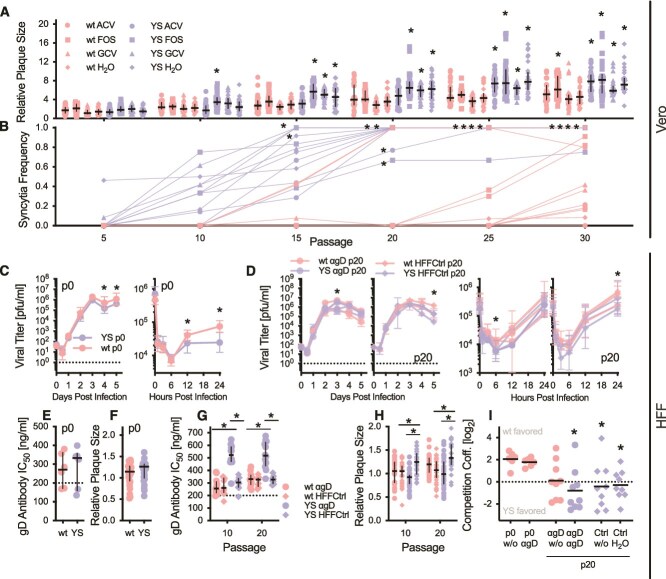
Rapid evolution of syncytial plaque phenotypes and antibody resistance in YS hypermutator. (A) Relative plaque sizes of on Vero cells evolved viruses. To passage 0 wt normalized areas were converted into diameters and used for the graph. Depicted are 30 plaques from three replicate populations (10 per replicate) as well as median and interquartile range. Similar data is displayed for HFF cells at passage 0 (F) and for passage 10 and 20 (H). These viruses were plated on HFF cells. * indicates significant differences (*P* < .05) measured by 2-way ANOVA followed by Dunnett’s (a, against wt H_2_O) and Tukey’s (H, as indicated) multiple comparison test, respectively. (B) Frequency of syncytial plaques throughout the passaging experiment on Vero cells. * indicates significant differences (*P* < .05) against wt H_2_O measured by 2-way ANOVA followed by Dunnett’s multiple comparison test. Growth curves on HFF cells for passage 0 (C) and passage 20 populations (D). Both multi-step (MOI 0.001, left) and single-step (MOI 10, right) growth kinetics were performed. * indicated significant differences (*P* <.05) observed *via* 2-way ANOVA and Šidák’s multiple comparison test. Resistance against αgD antibody measured by serum neutralization tests for passage 0 (E) as well as passage 10 and 20 (G). Displayed are 6 independent measurements per populations for passage 0 as well as 12 for passage 10 and 20 (4 per replicate population), respectively. Dashed lines indicate concentrations chosen for antibody selection. * indicates significant differences (*P* <.05) measured by 2-way ANOVA followed by Tukey’s multiple comparison test. (I) Competition assays for passage 0 and evolved passage 20 populations on HFF cells. Log_2_ transformed competition coefficients (number of wt genomes/number of YS genomes) were determined *via* qPCR for all possible wt/YS combinations (n = 9) in duplicates. * indicates significant differences (*P* <.05) against p0 w/o competition measured by 1-way ANOVA followed by Dunnett’s multiple comparison test.

Genotype phenotype correlations were performed by correlating SNP allele frequencies (Supplementary Table 2) with corresponding syncytia frequencies for the respective lineage (Fig. 1B). Strong and significant positive correlating variants (r ≥  0.9; *P*  < 0 .05) were arranged by their gene locus and displayed as variants per 1 kb of gene (Fig. 3A). Mutations that correlated in some lineages but didn’t in others were excluded.

### BAC mutagenesis and reverse genetics

To reverse engineer viral mutations, *en passant* mutagenesis in *E. coli* was utilized (Tischer *et al*. 2010) as described in earlier studies (Brunialti *et al*. 2023, Höfler *et al*. 2024). Mutagenesis primers can be found in Supplementary Table 1.

### Statistical analysis

All statistics given in this study were performed in GraphPad Prism v.9.4.0. For further information regarding specific tests, please see the respective figure legends.

## Results

### YS hypermutators are quickly adapting to cell culture conditions and antibody pressure

In a recently published study, we used a hypermutator HSV-1 mutant to study accelerated antiviral resistance development in Vero cell culture (Höfler *et al*. 2024). Whilst exploring evolution of antiviral resistance, we parallelly observed increases in plaque sizes in virus populations (Fig. 1A). Additionally, plaque phenotypes also shifted towards syncytia, which became the predominant plaque phenotype after 5–10 passages in the Pol^Y557S^ (YS) hypermutator (Fig. 1B). Importantly, those phenotypes evolved irrespective of the different selective pressures applied here and were neither promoted nor suppressed by antiviral treatment.

To increase our understanding of accelerated adaptation and expand our research into an environment comprised of more natural HSV-1 host cells, we passaged wt and YS hypermutator viruses on HFF cells in presence and absence of anti-gD antibody (αgD) pressure. Similar to previous results on Vero cells, wt and YS are growing with comparable kinetics on HFF cells, with only minor differences 4–5 days post infection (dpi) in multi-step growth curves (Fig. 1C, left). However, burst sizes are larger for wt in single-step growth curves (Fig. 1C, right). Initial differences in virus growth are largely compensated by passage 20 (Fig. 1D), particularly in single-step growth curves (Fig. 1D, right). Antibody resistance (Fig. 1E) and plaque sizes on HFF cells (Fig. 1F) are similar for passage 0 viruses, whereas after 10 and 20 passages, respectively, YS αgD display significantly increased antibody resistance (Fig. 1G). Plaque sizes increase in YS control populations only, whilst YS αgD tended towards smaller plaques (Fig. 1H). Initial competitive advantages observed in favour of wt completely disappear after 20 passages of selection on HFF cells, whereas antibody adaptation of YS became apparent only under αgD pressure (Fig. 1I).

### Glycoproteins evolve rapidly under multiple selective conditions

To understand genetic mechanisms underlying the phenotypes observed here, we performed whole genome sequencing of viral populations studied above and traced genetic changes across passages and under different selective pressures. In agreement with our observations for viral populations evolved on Vero cells (Höfler *et al*. 2024), genomes extracted from YS passaged on HFF cells contain around twice as many single nucleotide polymorphisms (SNPs) as wt (Supplementary Fig. 1A). Furthermore, evolutionary space is explored more rapidly by YS populations, as exemplified by 2-dimensional principal component analysis of per gene non-synonymous to synonymous substitution rates (dN/dS; Supplementary Fig. 1B, distances shown in Supplementary Fig. 1C). In line with the more natural environment for HSV-1, less genes feature mutations upon HFF selections, when compared to Vero cell selection (Supplementary Fig. 1D) (Höfler *et al*. 2024). Collectively, these results are in excellent agreement with our previous data on Vero cell derived HSV-1 populations.

**Figure 2:**
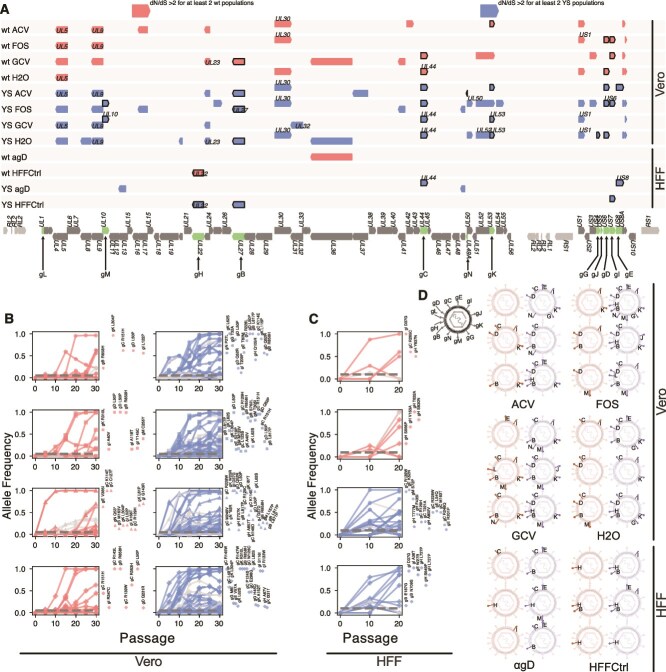
Genomic changes in glycoprotein genes upon Vero cell adaptation and gD antibody pressure. (A) Open reading frames (ORFs) under positive selection (dN/dS ratios above 2 for at least 2 out of 3 replicate populations) after multiple Vero cell adaptations as well as after HFF cell passages with and without αgD antibody pressure. Labelled ORFs indicates genes with dN/dS ratios above 2 for all three replicate populations. Framed ORFs mark glycoprotein genes. Individual SNP allele frequencies over passaging time for populations evolved on Vero cells (B) and HFF cells (C). Curves in grey and colour signify synonymous and non-synonymous changes, respectively. Dotted grey lines indicate the limit of detection (0.05 for Vero and 0.1 for HFF selections). Non-synonymous variants detected at passage 30 are labelled next to the respective plot at the corresponding end-point (p30 for B and p20 for C) allele frequency. (D) Map of glycoproteins affected by non-synonymous changes per replicate populations at endpoint passage.

We identified many genes under positive selection (defined by dN/dS ratios above 2 for at least 2 out of 3 replicate populations) upon Vero cell passaging, whilst HFF passaging yields fewer selected genes (Fig. 2A). Many of the genetic variants identified here affect glycoproteins. Specifically, positive selection is evident for genes encoding gD, gC, gK and gB in Vero and gE, gC, and gH in HFF cells. Genetic screening across our passaging experiment confirms that many glycoprotein mutations increase their allele frequency over time and become fixed in the population (Fig. 2B and C). Strong and uniform selection of specific glycoproteins becomes evident in replicate populations that show consistent affects across biological replicates (e.g. gC and gE in YS αgD populations; Fig. 2D).

### Mutations in glycoproteins gB, gD, and gK facilitate syncytia formation in Vero cell culture

**Figure 3:**
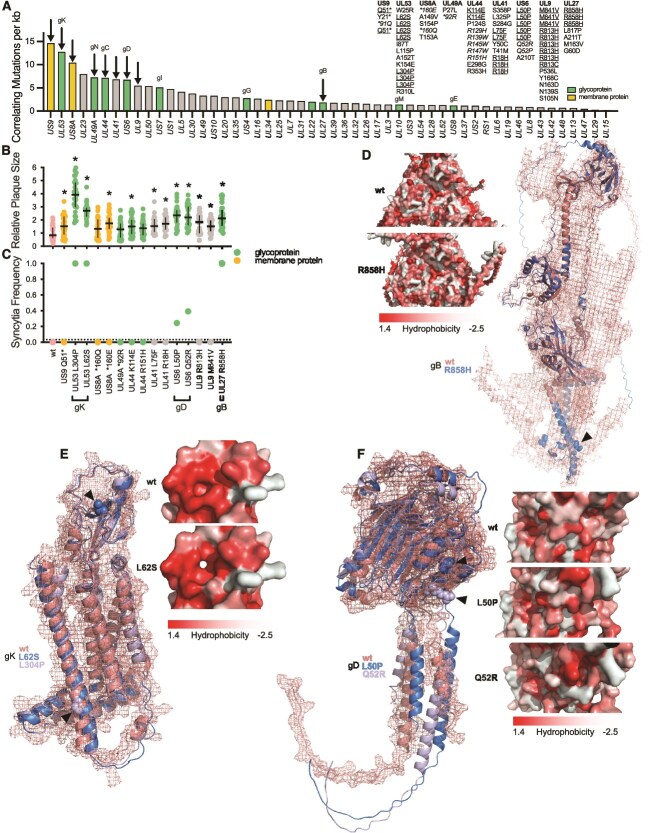
Glycoprotein mutations facilitate syncytia formation on Vero cells. (A) Genetic changes that correlate with syncytia frequency. Arrows mark genes we focused on. The table on the right shows individual SNPs, mutations that occurred multiple times independently are underlined whereas non-identical but similar changes are italicized. Relative plaque sizes (B) and syncytia frequencies (C) for reverse engineered mutants are displayed for 30 plaques + median and interquartile range. * indicates significant differences (*P* <.05) against wt measured by 1-way ANOVA followed by Dunnett’s multiple comparison test. Alphafold3 predictions for gB (D), gK (E), and gD (F) are displayed in overall structural alignments as well as surface changes. Mutated amino acids are displayed as dots and pointed towards with arrows.

Membrane fusion is an essential step in the life cycle of enveloped viruses. Therefore, viruses need to encode at least one fusogenic envelope protein, often a glycoprotein. In many viruses, syncytia formation occurs as a result of the fusogenic potential of those essential proteins. For individual lineages, we correlated allele frequencies of isolated genetic variants (Supplementary Table 2) with incidence of syncytia formation in the lineage (Fig. 1B) to identify specific mutations that may enhance cell fusion (Fig. 3A, see *Material and Methods* for more information). Our large dataset obtained from viruses facing different selective environments allowed us to focus on variants that independently occur in multiple lineages. Applying this strategy, we reduced the number of targets to 15 non-synonymous variants highly correlated which increased syncytia formation and occurring independently in multiple lineages. Next, we reverse engineered these mutations individually in the parental HSV-1 wt BAC to test their involvement in syncytia formation. We found that nearly all mutations lead to increased plaque sizes (Fig. 3B). However, only 5 amino acid changes indeed cause syncytia formation: L304P and L62S in gK, R858H in gB and, albeit to a lesser extent, L50P and Q52R in gD (Fig. 3C, plaques depicted in Supplementary Fig. 2).

To better understand and visualize the structural impact those amino acid changes might have on a protein level, we performed alphafold3 predictions (Abramson *et al*. 2024) and aligned mutant proteins with wt counterparts. Experimentally determined structures are available for both, gD and gB, and are in excellent agreement with our alphafold predictions (Supplementary Fig. 3A and B). However, as the structure of intrinsically disordered regions are notoriously difficult to predict, *in silico* modelling only allows for crude approximations of biological significance in those domains. As gB is a trimeric protein, we predicted the structure of the complete wt and mutant complex (Fig. 3D) to visualize impacts on quaternary structure. These predictions suggest that R858H might impact gB structure by changing the triangular head observed in the wt into a circular structure in the mutant. Additionally, the alphafold model only predicts the post-fusion conformation of gB, preventing us from drawing any conclusion on the pre-fusion state. For gK, L62S appears to impact the proteins head domain by forming a cavity and breaking a hydrophobic ring present in the wt protein. In the same protein, L304P may introduce a structurally subtler change by tilting (or probably breaking) the last transmembrane helix (Fig. 3E). Both gD mutants that affected syncytia formation (L50P and Q52R) appear to change surface structures in the head and might tilt transmembrane helices (Fig. 3F).

### High multiplicity of infection allows for syncytia formation upon cell entry

**Figure 4:**
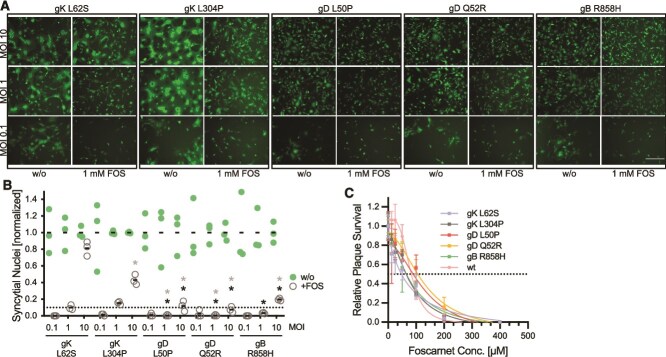
gK mutants engage in collective syncytia formation independently from genome replication. (A) Vero cells infected with glycoprotein mutants at different multiplicities of infection (MOI), with or without foscarnet (FOS) treatment. Pictures were taken with an inverted Zeiss Axio Vert.A1 fluorescence microscope at 100× magnification. The EGFP marker featured in the BAC backbone was utilized for visualization. Scale bar marks 100 μm. (B) Normalized counts of nuclei within syncytia from three independent infections. Filled green dots indicate infections without FOS whereas grey circles signify infections with FOS. * indicates significant differences (*P* <.05) measured by 2-way ANOVA followed by Tukey’s multiple comparison test. Black and grey * indicate significance (*P* <.05) against gK L304P and L62S, respectively, at the indicated MOI. (C) Plaque reduction assays for glycoprotein mutants against FOS. Dotted line indicates 50% survival.

Since we discovered multiple amino acid changes in different glycoproteins that facilitate syncytia formation, we set out to further characterize phenotypes of cell fusion within that subset. In principle, cell-to-cell fusion could occur during viral entry *via* virus mediated cross linking of multiple cells, or upon viral egress when glycoproteins are transported to the cell membrane. Although widely studied, it remains unclear at which step syncytia are generally formed. To distinguish between those two possibilities, we investigated syncytia formation at different multiplicities of infection (MOIs) with and without foscarnet (FOS) treatment. FOS acts as a direct inhibitor of the viral DNA polymerase and thus prevents replication, virion assembly and egress. Consequently, syncytia formation observed under high-dose FOS treatment could be attributed to viral entry. Indeed, we found that high MOIs cause little syncytia formation upon entry in all mutants studied by us. However, gK mutants display a substantial degree of apparently entry mediated syncytia formation which occurs despite complete abolishment of egress and plaque formation under FOS treatment (Fig. 4A–C).

### Increased particle stability and superinfection exclusion contributes to selective advantages of gK mutants

**Figure 5:**
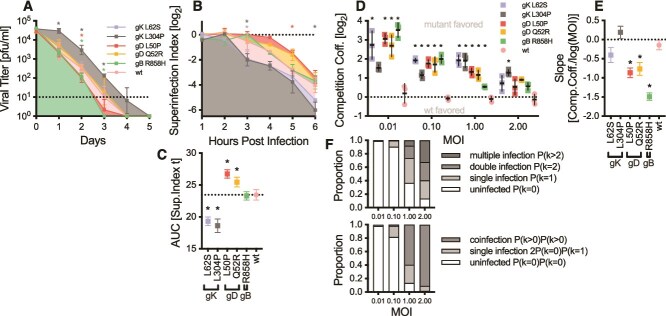
Advantages for gK mutants in particle stability, superinfection exclusion and competitions at increasing MOIs. (A) Survival curve for glycoprotein mutants in media. Dotted line indicates limit of detection. Coloured * indicate significant differences (*P* <.05) of the respective mutant against wt determined by 2-way ANOVA followed by Dunnett’s multiple comparison test. (B) Superinfection exclusion measured by superinfecting glycoprotein mutants with mCherry labelled wt at indicated timepoints post infection. Superinfection index (mCherry plaques/EGFP plaques) were log_2_ transformed and plotted over time. Dotted line shows no superinfection exclusion. Coloured * indicate significant differences (*P* <.05) of the respective mutant against wt determined by 2-way ANOVA followed by Dunnett’s multiple comparison test. (C) Area under the curve (AUC) of superinfection exclusion data from (B). Dotted line signifies wt levels. * indicates significant difference (*P* <.05) to wt. (D) Competition assays of glycoproteins against wt at different MOIs. * indicates significant differences against wt/wt competition measured by 2-way ANOVA followed by Dunnett’s multiple comparison test. (E) Slope of data from (D) calculated by linear regression. * indicates significant difference (*P* <.05) to a slope of 0. (F) Proportions of uninfected, single infected, double infected, and multiple infected (upper) as well as uninfected, single infected, and coinfected (lower) cells for single genotype and double genotype infections at indicated MOIs, respectively.

Next, we aimed at uncovering further phenotypic differences conferred by syncytial virus variants. Since glycoproteins are important structural elements of virions and determinants of infectivity, we investigated the effect of the above-described mutants on viral infectivity over time. Exposing virions to prolonged incubation in growth media (see *Material and Methods*), we found that gK L304P appears to confer a significant stability advantage and endows virus particles with prolonged infectivity. Contrarily, gD L50P and gB R858H appear to be less stable than wt and show a faster decay in viral titers (Fig. 5A). Apart from stability, we also investigated the ability of mutants to exclude superinfecting wt virus from Vero cells. Interestingly, superinfection exclusion (SIE) seems to be somewhat independent of syncytia formation, as both gK mutants exclude superinfecting virus significantly faster whilst gD mutants are more susceptible to superinfection (Fig. 5B and C). Collectively, these findings suggest potential trade-offs and implications of syncytia formation, especially for gK L304P.

To assert selective advantages conferred by gK variants, we performed competition assays against mCherry tagged wt virus at different MOIs. As expected, all mutants have a significant advantage and are outcompeting wt virus (Fig. 5D). Interestingly, competition coefficients are decreasing with increasing MOI, indicated by negative slopes observed in competition curves (Fig. 5E). However, gK variants exhibit fitness advantages at all tested MOIs. L304P maintains a significant competitive advantage over wt across all tested MOIs, whilst L62S shows a stable advantage for MOIs of 0.01, 0.1, and 1, however, decreases to gD and gB mutant levels at a MOI of 2. As likelihood of infection and coinfection of cells drastically increases at higher MOIs (Fig. 5F), the stable advantage of gK variants over wt across rising MOIs suggests some protection against coinfecting particles and genotypes.

### Resistance against neutralizing gD antibody is mediated by gE in a collectively beneficial manner

**Figure 6:**
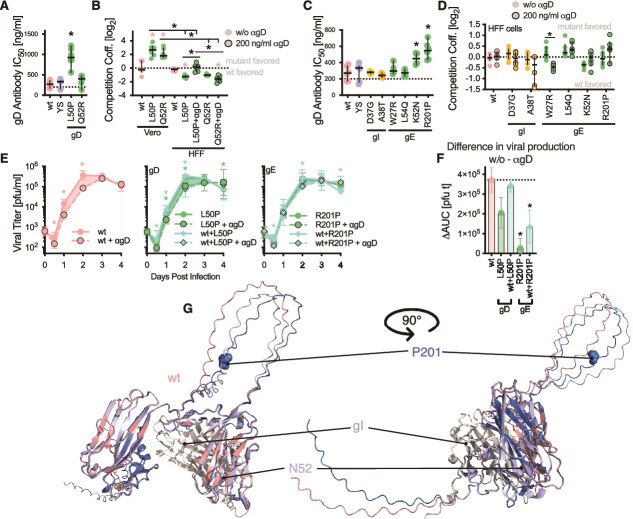
gD antibody resistance is mediated by gE mutations, which rescue wt virus. Antibody resistance measured by serum neutralization tests for wt, YS as well as gD (A) and gI/gE mutants (C). IC_50_ values are given for 6 independent dilution series, depicted with median and interquartile range. * indicates significant difference (*P* <.05) to wt measured by 1-way ANOVA followed by Dunnett’s multiple comparison test. Competition assays for gD (B) and gI/gE mutants (D). * indicate significant difference (*P* <.05) as indicated (red * against wt/wt competition) measured by 1-way ANOVA followed by Tukey’s (B) as well as 2-way ANOVA followed by Šidák’s (D) multiple comparison tests, respectively. (E) Multi-step growth curves (MOI 0.01) for wt, gD, gE, wt + gD, and wt + gE populations with and without gD antibody pressure. * indicates significant difference (*P* <.05) between curves with and without antibody treatment, measured by 2-way ANOVA followed by Tukey’s multiple comparison test. (F) Difference between non-treated and treated area under the curve for data from E (coloured areas). * indicate significant differences (*P* <.05) in antibody inhibition. (G) Alphafold3 structure predictions for gI/E glycoprotein complexes. Structure of gE is given in coloured cartoon configuration (red for wt, blue for R201P and light blue for K52N). gI is presented in grey whilst single mutations (R201P and K52N) are shown as spheres.

Immune evasion is an essential mechanism of viral survival and persistence (Vossen *et al*. 2002). Immune escape mutants that, for example, evade specific neutralizing antibodies, are therefore of special importance. To study immune escape, we selected for gD antibody resistance by passaging HSV-1 wt and YS hypermutator in the presence of monoclonal murine αgD derived from the potently neutralizing human clone E317 (Lee *et al*. 2013) on HFF cells. Contrarily to what we expected, we failed to detect mutations in *US6* (gD), even after 20 passages of αgD treatment. However, we evaluated gD variants previously evolved on Vero cells for antibody resistance. Indeed, gD L50P significantly increased resistance against αgD (Fig. 6A). To investigate why gD mutants were not selected on HFF cells, despite conferred resistance to αgD, we performed competition assays on both cell lines. We find that on Vero cells, gD mutants are positively selected, whereas they are outcompeted by wt on HFF cells (Fig. 6B). Even αgD resistance mediated by gD L50P is not enough to overcome the intrinsic fitness cost of this mutant on HFF cells, arguing for evolutionary constrains on gD receptor recognition. Higher antibody concentrations, however, might result in scenarios in which gD mediated resistance is more beneficial than the associated cost.

Even though there was an absence of changes in the epitope targeted by the antibody, we did observe rapid protein evolution and positive selection in *US8* (gE; Fig. 2A). Resistance against αgD increased in gE K52N and R201P, but not in other gE or any gI mutants (Fig. 6C). Both of those mutants fail to exhibit significant advantages over wt upon antibody treatment in competition assays (Fig. 6D). To further explore resistance profiles of our gE mutants, we performed multi-step growth kinetics in presence and absence of αgD. Whilst wt, gD L50P as well as a mixture of both (wt + L50P) were significantly inhibited by αgD treatment, gE R201P and the wt + R201P mixture both grew significantly better and similarly well despite αgD treatment (Fig. 6E). Area under the curve transformation of that data confirms this observation. We calculated an ‘area between the curves’ to illustrate the differences in growth in presence and absence of antibodies (Fig. 6F). A larger titer difference in this graph indicates stronger suppression by αgD. Since wt is not outcompeted by resistant gE mutants and wt growth is rescued by gE R201P, it is tempting to speculate that the occurrence of this mutation in the population presents an advantage not only for viruses carrying the mutation, but also other members of the population. This speculation is further supported by the low allele frequency of R201P in YS αgD populations (~0.1; Fig. 2C). Mutually beneficial interactions between co-infecting genotypes can lead to negative frequency dependent selection as shown for influenza viruses, hepatitis C and in theoretical models (Skums *et al*. 2015, Xue *et al*. 2016, Leeks *et al*. 2018). Both gE amino acid changes occur within the ectodomain of the gI/E heterodimer as shown by our alphafold3 predictions and in experimentally determined structures (Fig. 6G, Supplementary Fig. 3C), a position known to act as a Fc receptor (Baucke and Spear 1979, Dubin *et al*. 1991). R201P maps to an intrinsically disordered domain, a region which is difficult to predict, however, such regions have been shown to be important for protein–protein interactions (Uversky 2018).

## Discussion

Surface glycoproteins belong to the most rapidly evolving proteins of viruses (Schulze and Manger 1992, Elder *et al*. 1977, Shamblin *et al*. 2004, Lamers *et al*. 2015). Essential for host-cell attachment and entry, whilst at the same time exposed to humoral and cellular immune responses, surface proteins are under immense evolutionary pressure (Vossen *et al*. 2002, Thomson *et al*. 2021). HSV-1 virions feature 12 glyco- and 5 membrane proteins, making the viral envelope a highly diverse and complex microenvironment (Hilterbrand and Heldwein 2019). In this study we discovered HSV-1 glycoprotein variants that mediate phenotypes beneficial to *in vitro* evolved viral populations.

We recently established a mild hypermutator virus to accelerate experimental evolution of HSV-1 (Höfler *et al*. 2024). In our previous work, we used Vero cell culture to study antiviral resistance development. However, upon Vero cell passage, we also observed increased plaque sizes and syncytial phenotypes in evolved populations. Syncytia are in many ways beneficial to *in vitro* evolved viruses and thus rapidly selected in cell culture (Symeonides *et al*. 2015, Kuny *et al*. 2020). Primarily, syncytia allow for more efficient cell to cell spread (Jessie and Dobrovolny 2021). This, in turn, allows infecting viruses to better exploit cellular resources and gives them selective advantages, resulting in their rapid fixation. Also, higher fusogenic potential decreases attachment and entry time, facilitating faster infection and initiation of genome replication (Tang *et al*. 2019). However, there is a cost to syncytia formation: syncytia associated apoptosis limits selective advantages by shortening cellular survival which may reduce formation of infectious viral progeny (Ferri *et al*. 2000, Ma *et al*. 2021). Despite this, beneficial aspects of syncytia formation appear to generally outweigh deleterious ones, resulting in selection for highly fusogenic particles.

Whilst only four out of 15 reverse engineered mutations enabled syncytia formation, it is conceivable that a combination of multiple mutations, potentially located on different genomes, could also rescue the phenotype. Such observations are reported for Measles virus, where mutations located on the same genome had negative effects on fusogenicity. However, spread across different genomes, these same mutations did facilitate syncytia formation (Shirogane *et al*. 2023).

Only four glycoproteins are known as essential for HSV-1 attachment and entry, namely gD, gH, gL, and gB (Hilterbrand *et al*. 2021). We identified syncytial mutations in two out of those four essential glycoproteins. Additionally, we found gK mutants to mediate hyper-fusiogenic behaviour. In agreement with our findings, gK is described as involved in cell fusion (Hutchinson *et al*. 1992). Evidently, interactions between gK and other glycoproteins alter cell fusion, an interplay in which gK plays an inhibitory role (Avitabile *et al*. 2003). Interestingly our results show that only gK mutants displayed any significant ability to promote syncytia formation upon viral entry. This may be due to high local concentrations of gK in close proximity to gB on the particles surface, where gK/gB interaction can inhibit fusogenic activity of gB in wt particles (Jambunathan *et al*. 2011). One possible interpretation of our data would suggest that upon egress, dilution of viral glycoproteins across the cell surface may abolish gK’s local effect on gB inhibition. Additionally, gK dependent cell fusion upon viral entry is highly influenced by MOI, as viral particles are the limiting factor in this scenario and only simultaneous infection of neighbouring cells produces syncytia.

Strikingly, gK also appears to be critical for particle stability. gK variants observed by us endowed virions with significantly prolonged infectivity under typical cell culture conditions. This phenomenon is most likely due to higher association to cellular membranes which protect virions from initial degradation (Musarrat *et al*. 2018, Rider *et al*. 2019). Furthermore, our gK variants initiated earlier SIE. From 3 h post infection on, gK mutants significantly decrease reproduction of superinfecting wt virus. Whether this is due to faster attachment and cell entry, more efficient cell to cell fusion, or the presence of mutant gK on cellular surfaces, is difficult to untangle. Since other syncytial mutants do not accelerate SIE, we do not directly ascribe SIE to a syncytial phenotype. However, early syncytia formation as observed in gK mutants may contribute to the observed increase in SIE (Musarrat *et al*. 2018). SIE in HSV-1 is dependent on viral gene expression, and specifically on downregulation and shutoff of the cellular receptors (Stiles *et al*. 2008, 2010, Criddle *et al*. 2016, Friedel Caroline *et al*. 2021). Syncytia formation induced by gK mutants might increase the number of infected cells simply by fusion of infected and uninfected cells, rendering a higher percentage of cells refractory to superinfection. In agreement with this hypothesis, gK mutants maintain their fitness advantages with increasing MOIs. In other mutants, specifically gB R858H, syncytia formation depends on viral genome replication and egress. Increasing MOI diminishes their ability to outperform wt, as viral spread is less important under conditions of high MOI. gK mutants, however, which also form syncytia upon cell entry, still excel at higher MOIs, suggesting an advantage conferred by their ability to exclude superinfecting particles from cells occupied by them.

With humans as reservoir host (Reichman 1984, Everett 2014), HSV-1 is expected to be well adapted to human cells and experience comparatively little change upon culture in such. Indeed, compared to the notable diversity of proteins under positive selection in Vero cells, HFF cells provide less adaptive genome space, leading to fewer mutations, purifying selection and slower protein evolution. However, antibody pressure clearly led to positive selection of gE and gC (possibly even some co-evolution), at least in YS hypermutator populations. Those populations also display selection for *UL13* and *UL49*, although to a lesser degree. Nevertheless, mutations in these genes could be relevant to the observed increase in antibody resistance. *UL49* encodes VP22, a tegument protein important for neurovirulence and effective cell-to-cell spread (Brignati *et al*. 2003, Yu *et al*. 2010, Tanaka *et al*. 2012). Antibody treatment may select for intracellular viral phenotypes which remain inaccessible to neutralizing antibodies throughout the viral life cycle. *UL13* on the other hand encodes a protein kinase phosphorylating numerous cellular and viral proteins (e.g*.* VP22), hitchhiking regulatory networks in the infected cell (Kawaguchi *et al*. 2003, Asai *et al*. 2007), which may be selected as a consequence of antibody pressure. Except for *UL36*, no convergently selected genes appear in wt populations upon antibody exposure, this finding is consistent with the observed absence of any antibody resistance in wt populations. Other than that, gH mutants seem to be selected for under relaxed conditions, probably enhancing the attachment and entry as the mediator protein. Surprisingly, antibody treatment suppressed any selection of gH mutants. This could be due to an overall lower genetic diversity observed in viral populations evolved on HFF cells, which limits the pool of genotypes available to selection. High selection pressure for antibody evasion might have interfered with gH selection because of limited mutational input. Another possible explanation could be the exposure of conformational epitopes in gD upon mutant gH binding and henceforth enhanced antibody recognition (Mortimer and Minchin 2016). Since plaque sizes do only increase in the absence of antibody pressure, there might be a gH mediated trade-off between cell-to-cell spread and antibody resistance.

In revealing paths to antibody resistance within an evolutionary extremely short time frame, our YS hypermutator, again, proves its outstanding ability to accelerate evolutionary processes by providing extended genomic sequence space which is inaccessible to wt (Xing *et al*. 2022, Höfler *et al*. 2024). This ability of our hypermutator virus to shorten observation periods will likely be useful in future work on experimental evolution of HSV-1, and possibly other herpesviruses in which homologous mutations confer similar effects (Trimpert and Osterrieder 2019, Xing *et al*. 2022).

Our *in vitro* selection of YS hypermutator populations for gD antibody resistance suggested multiple targets for resistance development. As gE is known to act as a Fc receptor (Dubin *et al*. 1991), and gC as well as UL13 are established interaction partners of gE (Ng *et al*. 1998, Hook *et al*. 2008), we decided to concentrate on gE mutations and reverse engineered them along with gI mutations. We find that indeed, gE mutations K52N and R201P significantly increase resistance against αgD. Both mutations occur exclusively in YS populations selected for αgD resistance. Initially, we found the absence of gD mutations in those populations puzzling, however, resistant gD substitutions, independently selected in absence of antibody pressure on Vero cells, exhibited fitness costs *in vitro* in HFF cells. Opposing selection pressures for sustained or increased receptor binding and antibody evasion potentially led to gene capture events or duplications. This might have enabled ancestral herpesviruses to bind antibodies *via* Fc receptors, endowing them with a significant selective advantage (Gibson and Spear 1983, Elde *et al*. 2012, Gao *et al*. 2017, Brito and Pinney 2020). Indeed, gene captures of immune modulatory genes are described for HSV-1 (Schönrich *et al*. 2017). As part of a human virus, HSV-1’s gE evolved to bind human Fc domains (Ndjamen *et al*. 2014). However, since we used a mouse monoclonal IgG antibody in this study, gE may have rapidly evolved to accommodate binding of mouse Fc and circumvent antibody pressure. It is tempting to speculate that, had we used human antibodies or only Fab fragments, we might had selected for gD or even gH/L variants that allow immune evasion. Adaptation to mouse Fc also provides insights into how host spillovers in herpesviruses can occur, a process frequently overlooked in their evolution (Brito *et al*. 2021). Overcoming the immune response mounted by a novel host is an essential step to successfully establish reliable transmission within new host species (Plowright *et al*. 2017). Remarkably, gE mediated antibody binding appears to be beneficial for other co-infecting genotypes, shielding them from neutralization in a social manner. Whilst our results demonstrate social interaction within our viral populations, more work is needed to fully describe the costs and benefits of such interactions and to uncover underlying biological mechanisms. Indeed, social interactions within viral communities are frequently overlooked, but play important roles in the life cycles of many viruses (Díaz-Muñoz *et al*. 2017, Sanjuán 2021, Leeks *et al*. 2023b). Both, syncytia formation and Fc antibody binding, may be considered social phenotypes. Syncytia enable manifold intracellular interactions between viruses in neighbouring cells (Steain *et al*. 2008), which also expands the spatial scale of public goods use (Chao and Elena 2017). Similar phenotypes have been observed for multipartite plant viruses which share proteins across cells (Sicard *et al*. 2019). This type of interaction would also benefit defective viral genomes, which cannot independently establish productive infection (Rezelj *et al*. 2018, Vignuzzi and López 2019). Syncytia might influence virion aggregation and allow for collective transmission of defective genomes (Andreu-Moreno and Sanjuán 2020). Similarly, Fc receptors such as gI/E bind antibodies, thus clearing them from the extracellular environment and enabling all members of the viral population to infect susceptible cells irrespective of their gI/E genotype (Dubin *et al*. 1991, Ndjamen *et al*. 2014). More and more social interactions within and between viral populations have been discovered in recent years (Díaz-Muñoz *et al*. 2017, Sanjuán 2021, Haney *et al*. 2022, Leeks *et al*. 2023a, 2023b). Just as molecular pathways and interactions are driven by evolution, population dynamic and social interactions drive viral evolution in response to specific selective pressures. Future research should be directed at further elucidation of novel aspects in sociovirology.

A limitation of this study is presented by the singular focus on cell culture systems. Whilst HFF cells are human, HSV-1 does not primarily infect fibroblasts in a natural infection setting (Howley *et al*. 2021). Vero cells are neither derived from a natural host, nor are they a tissue and are furthermore defective in the type I interferon response (Emeny and Morgan 1979). However, this apparent disadvantage may prove useful when considering spillover events. Many spillovers require immunosuppressed initial hosts to accumulate essential adaptations (Bean *et al*. 2013, Weigang *et al*. 2021, Warren *et al*. 2022, Li *et al*. 2023). In this context, Vero cells might provide a valuable environment to mimic some aspects of host spillovers under relaxed immunological conditions. Specifically, the rapid evolution of glycoproteins upon Vero cell selection argues towards their importance for exploring new hosts. gD mutants which confer selective advantages on Vero cells are deleterious on HFFs, suggesting improved foreign receptor recognition or exploration of novel host cell receptors, which are likely encountered upon host spillovers. HSV-1 does occasionally infect other primates. Whilst infections of old world monkeys are usually well tolerated (McClure *et al*. 1980, Eberle and Hilliard 1989), they are often fatal in new world monkeys (Huemer *et al*. 2002). Vero cells are derived from *Chlorocebus sabaeus*, an old world monkey species. Given the evolutionary distance between those two clades, immune recognition might be an important determinant of virulence. Another major aspect of pathogenicity in non-host species might be the complement system (Huemer *et al*. 1993), usually antagonized by HSV-1 *via* gC interactions (Friedman *et al*. 1984). More work is required to fully understand herpesvirus host spillovers.

Overall our study contributed to the already known diversity of glycoprotein functions in HSV-1 and elucidated novel aspects of their evolution. In addition, we provide evidence for the advantage conferred by the use of a hypermutator virus in experimental evolution. Future studies will be aimed at advancing our understanding of glycoprotein variants and their role as immune modulatory factors, targets of viral interaction and pivotal effectors of viral attachment and entry.

## Supplementary Material

GlycoPaper_SupTables_veaf072

GlycoPaper_SupFigures_veaf072

## Data Availability

Used code and sequencing raw data are available at github (https://github.com/hoeflet/antiviral-resistance-evolution.git) and the NCBI SRA under BioProject accession number PRJNA927130, respectively.
