# Supplementary material for: Rapid glycoprotein evolution enables variant interactions in herpes simplex virus type 1
Source: Virus Evol. 2025 Sep 17;11(1):veaf072. doi: 10.1093/ve/veaf072 (PMC12499918; doi:10.1093/ve/veaf072)
Supplement: GlycoPaper_SupFigures_veaf072 [file glycopaper_supfigures_veaf072.pdf]

Rapid Glycoprotein Evolution Enables Variant Interactions in Herpes Simplex Virus Type 1

Thomas Höfler[1,2], Michaela Zeitlow[1], Ji Yoon Kim[1,2], Emanuel Wyler[3] & Jakob Trimpert[1,2#]

[1] Institut für Virologie, Fachbereich Veterinärmedizin, Freie Universität Berlin, Berlin, Germany

[2] Department of Diagnostic Medicine and Pathobiology, College of Veterinary Medicine, Kansas State University, Manhattan, KS, USA

[3] Berlin Institute for Medical Systems Biology, Max-Delbrück-Center for Molecular Medicine in the Helmholtz Association, Berlin, Germany

[#] corresponding author: jtrimpert@vet.k-state.edu

Supplementary Figures

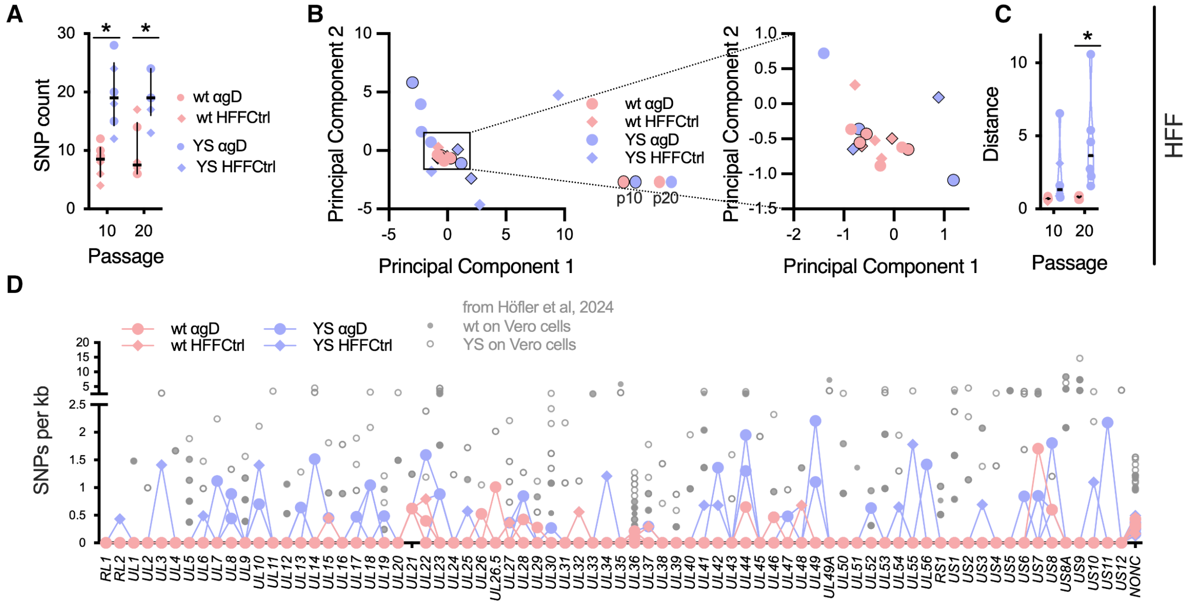

Sup. Figure 1: Higher mutation frequency of YS mediates faster evolutionary movement. A) SNP counts for on HFF cells evolved wt and YS populations. * indicates significant differences measured by 2-way ANOVA followed by Šidák's multiple comparison test. **B)** 2-dimensional principle component analysis for non-synonymous to synonymous substitution rates (dN/dS). Full set (left) was zoomed in to highlight less diverse samples (right). **C)** PCA distances for points in (**B**). * indicates significant differences measured by 2-way ANOVA followed by Šidák's multiple comparison test. **D)** SNPs detected at least once throughout the passaging experiment are plotted according to their genomic location. Red and blue symbols feature on HFF evolved wt and YS populations respectively, while grey dots and circles indicate on Vero cells evolved wt and YS populations, adapted from Höfler et al, 2024. Each grey dot and circle indicate SNPs per kb of the respective gene from a single lineage. Data derived from passaging on Vero cells under varying selection pressures. NONC stands for non-coding SNPs.

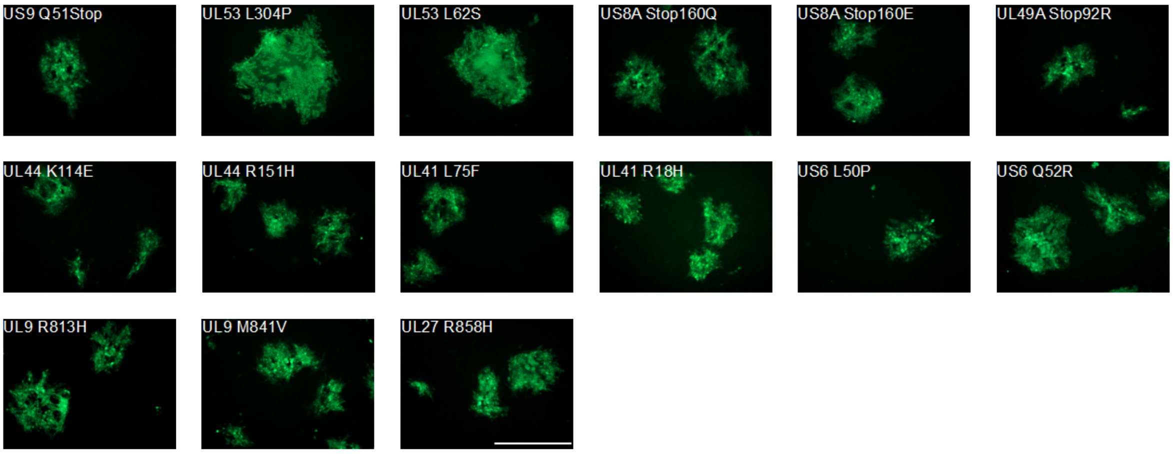

Sup. Figure 2: Plaque phenotypes for with syncytia frequency correlating mutants. Plaque pictures for reverse engineered mutants, taken 2 days after infecting a monolayer of Vero cells with an inverted Zeiss Axio Vert.A1 fluorescence microscope at 100x magnification. The EGFP marker featured in the BAC backbone was utilized for visualisation. Scale bar marks 500 µm.

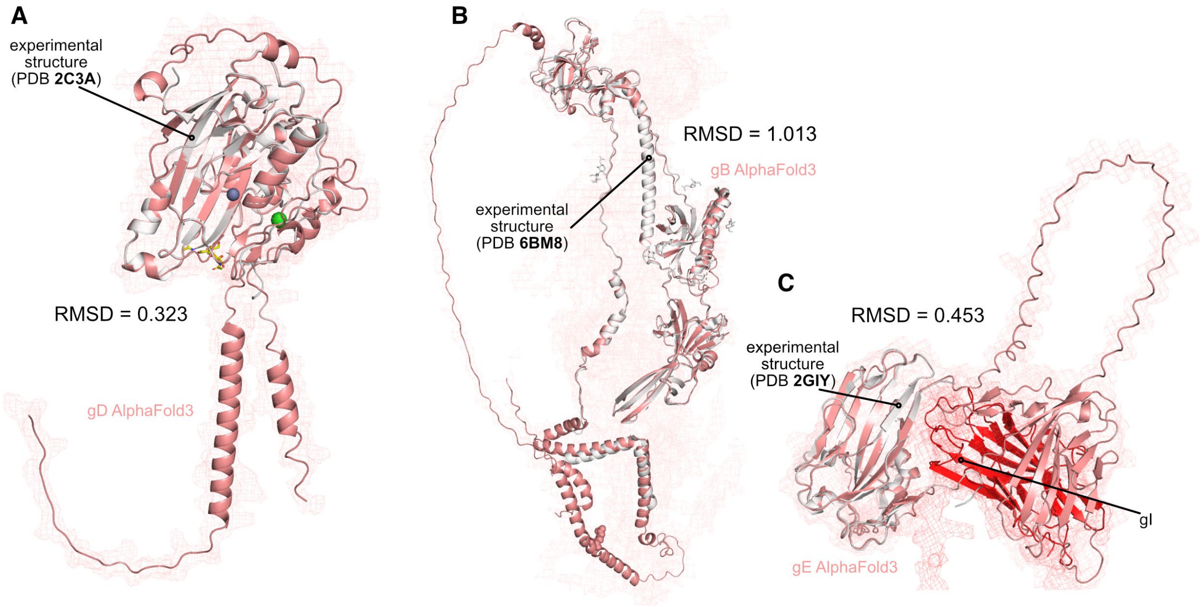

Sup. Figure 3: Structural alignments of experimentally determined glycoprotein structures with AlphaFold3 predictions. Experimental structures were downloaded from the protein data bank (PDB, individual accession numbers are stated boldly for the respective structures) and aligned with predicted AlphaFold3 wild type models using PyMOL. Overall atomic root mean square deviation (RMSD) is given for all performed alignments in Angstrom (Å). Experimental structures are given in white, AlphaFold3 predictions in red.
